# Electrical stimulation paradigms on muscle quality and bone mineral density after spinal cord injury

**DOI:** 10.1007/s00198-025-07482-5

**Published:** 2025-04-22

**Authors:** Ashraf S. Gorgey, Siddharth Venigalla, Jakob N. Deitrich, William B. Ballance, William Carter, Timothy Lavis, Robert A. Adler

**Affiliations:** 1 Spinal Cord Injury and Disorders, Richmond VA Medical Center, 1201 Broad Rock Blvd, Richmond, VA 23249 USA; 2 Physical Medicine and Rehabilitation, School of Medicine, Richmond, VA USA; 3 VCU-Sheltering Arms Institute, Richmond, VA USA; 4 Endocrinology Service, Central Virginia VA Healthcare System, Richmond, VA USA; 5 Endocrine Division, School of Medicine, Virginia Commonwealth University, Richmond, VA USA

**Keywords:** BMD, Muscle quality, Peak torque, Rehabilitation, SCI

## Abstract

**Summary:**

The goal of the work was to determine the effects of altering muscle quality (peak torque and muscle CSA) via NMES-RT on bone mineral density (BMD) following application of FES-lower extremity cycling. Components of muscle quality were altered and attenuated the decline in BMD after SCI.

**Introduction:**

Spinal cord injury (SCI) negatively impacts muscle quality and bone health. Neuromuscular electrical stimulation-resistance training (NMES-RT) has been shown to enhance muscle quality. It is unclear whether adding NMES-RT to functional electrical stimulation (FES)-lower extremity cycling may further augment muscle quality and subsequently enhance bone mineral density (BMD).

**Methods:**

Thirty-two participants were randomized into either 12 weeks of NMES-RT followed by 12 weeks of FES- lower extremity cycling (NMES-RT + FES; *n* = 16) or 12 weeks of passive movement training (PMT) followed by 12 weeks of FES-lower extremity cycling (PMT + FES; *n* = 16). Measurements were conducted at baseline (BL), post-interventions 1 and 2 (P1 and P2) separated evenly by 12 weeks. Left thigh muscle isometric and isokinetic torques were measured using an isokinetic dynamometer. Magnetic resonance imaging measured whole thigh and knee extensor (KE) muscle CSAs. Dual energy X-ray absorptiometry measured total and regional BMD.

**Results:**

NMES-RT elicited a trend towards greater isometric torque at 80 Hz (*P* = 0.057) and isokinetic torque (60 deg/s; *P* = 0.009 and 180 deg/s; *P* = 0.003) compared to PMT. Muscle CSA was greater in left whole thigh (F (2,20) = 9.1; *P* = 0.007) and KE (F (2,20) = 15.5; *P* = 0.001) by 11.0 and 8.0 cm^2^ respectively at P1 in the NMES-RT + FES compared to PMT + FES. In the NMES-RT + FES, ankle weights were positively associated with muscle CSA, isometric and isokinetic torques as well as muscle quality following P1. Compared to PMT + FES, NMES-RT + FES maintained BMD at the distal femur.

**Conclusion:**

NMES-RT + FES enhanced muscle quality as measured by torque production and muscle CSA as result of increasing ankle weights. The addition of FES- lower extremity cycling to NMES-RT maintained but did not further augment muscle quality. Furthermore, NMES-RT + FES may help maintain BMD after SCI.

**Clinical trial registration:**

Registered with clinicaltrials.gov: NCT02660073.

## Introduction

Skeletal muscle quality has previously been defined as the ratio of maximum peak torque relative to the cross-sectional area (CSA) of the target muscle group [1–3]. Decline in muscle quality has been considered as a critical index of age-related sarcopenia, impairment in physical functions and reduction in activities of daily living [4, 5]. Spinal cord injury (SCI) results in detrimental changes to musculoskeletal health including extreme skeletal muscle atrophy, fiber type transformation more fatigable fast twitch fibers and infiltration of intramuscular adipose tissue [6–10]. The transformation of morphological properties of the paralyzed muscles results in impairment of muscle quality and diminished tension production as measured by either isometric or isokinetic torque production [3]. Furthermore, a recent cross-sectional trial indicated impairment of passive stiffness of the paralyzed knee extensor muscle as result of infiltration of intramuscular adipose tissues [11]. The authors suggested a positive relationship between muscle CSA and passive stiffness as measured by magnetic resonance elastography [11]. It is possible to assume that the decrease in muscle quality after SCI is a triggering factor for development of neurogenic osteoporosis [8–10]. The latter is characterized by bone porosity, sub-lesion fractures and long-term hospitalization [9]. Therefore, rehabilitation strategies that augment muscle quality may further enhance or maintain bone health in persons with SCI.

Loading the paralyzed muscles via applications of surface neuromuscular electrical stimulation (NMES) robustly evokes skeletal muscle hypertrophy [3, 12, 13], increases mitochondrial density and activity, and enhances muscle quality after SCI [13]. Holman and Gorgey previously noted that NMES-resistance training (RT) with testosterone treatment enhanced muscle quality compared to testosterone treatment only in persons with SCI [3]. The authors noted that 16 weeks of NMES-RT increased both peak knee extensor (KE) isometric and isokinetic torques as well as muscle CSA in persons with SCI [3]. The findings demonstrated improvements in knee extensor contractile properties via increasing rise time and slowness of half-time to relaxation [3]. The same group suggested that the same protocol (NMES-RT + testosterone treatment) enhanced distal femur and proximal tibia bone microarchitecture as measured by magnetic resonance imaging (MRI) following 16 weeks of training [14]. The authors concluded that 16 weeks of open kinematic chain NMES-RT has augmented bone microarchitecture via enhancing muscle quality; however, this has not been tested.

Unlike NMES-RT, close-kinematic chain exercises via application of functional electrical stimulation (FES) are commonly prescribed to load the bone and augment bone mineral density after SCI [15–17]. FES-rowing was suggested as an intervention that attenuates time dependent changes in trabecular bone loss in persons with SCI [15]. Another study demonstrated that 90 FES-rowing exercise sessions over 9 to 12 months resulted in controversial findings [16]. Two participants experienced a substantial decrease and the remaining two demonstrated increases in the trabecular bone mineral density (BMD) at the distal femur and distal tibia [16]. The study noted that the loading cycle of the FES-rowing was correlated with the changes in the trabecular BMD of the distal femur [16]. Another study demonstrated positive changes in knee microarchitectures following 6 months of low-intensity FES-lower extremity cycling in children with SCI [17]. Therefore, it is unclear whether a rehabilitation strategy that combines both open and closed-kinematic chain exercises may induce additive beneficial effects on BMD after SCI.

Compared to the general population, the interplay between muscle quality and bone heath is not well studied after SCI [18, 19]. This is highly empirical to the rehabilitation of the SCI population considering the accelerating rate of bone loss and the high prevalence of neurogenic osteoporosis [8, 9]. A recent randomized clinical trial has explored the benefits of combining NMES-RT and FES- lower extremity cycling on cardio-metabolic risk factors [13]. The major finding of the study was that 12 weeks of NMES-RT robustly evoked muscle hypertrophy and the addition of FES- lower extremity cycling maintained the gain in muscle mass. It is plausible to assume that preceding FES- lower extremity cycling with NMES-RT would result in increased peak evoked torques of the knee extensor muscle group and impose greater tension on the bone to enhance bone mineral density (BMD) similar to what has been reported in the general population [18]. Therefore, we hypothesized that 12 weeks of NMES-RT followed by 12 weeks of FES- lower extremity cycling would augment muscle quality and subsequently enhance regional BMD in persons with SCI.

## Methods

Thirty-two participants with chronic (> 1-year post injury) SCI were randomized into either NMES-RT + FES (*n* = 16) or PMT + FES (*n* = 16; Table 1). Using block randomization, participants were randomized into either 12 weeks of NMES-RT followed by 12 weeks of FES-LEC or PMT for 12 weeks followed by 12 weeks of FES- lower extremity cycling. The entire duration of the study was 27 weeks (3 weeks of measurements and 24 weeks of training) [13]. Measurements were conducted at baseline (BL; prior to starting any intervention), post-intervention 1 (P1; 12 weeks after intervention) and post-intervention 2 (P2; 24 weeks after intervention).

**Table 1:** Physical and SCI characteristics at baseline of 32 individuals after randomization into NMES-RT + FES (*n* = 16) or PMT + FES (*n* = 16) for 24 weeks

	NMES-RT + FES (*n* = 16)	PMT + FES (*n* = 16)	***P-values***
Age (yrs.)	37.4 ± 12.5	42.0 ± 14.0	0.33
Body Weight (kg)	73.0 ± 16.5	68.3 ± 13.0	0.39
Height (m)	1.76 ± 0.08	1.74 ± 0.1	0.67
BMI (kg/m^2^)	24.0 ± 6.0	23.0 ± 4.0	0.52
Ethnicity	9 white/7AA	8 white/8 AA	
Time since injury (yrs)	11.5 ± 11.0	10 ± 11	0.66
Injury Class	11 Paraplegia: 5 Tetraplegia	11 Paraplegia: 5 Tetraplegia	
LOI	C5-T12	C5-L1	
AIS classification	8 AIS A: 5 AIS B: 3 AIC C	10 AIS A: 4 AIS B: 2 AIC C	
Number of Participants completed BL	16	16	
Number of Participants completed P1	14	16	
Number of Withdrawals	**2** - None compliant - Low BMD at P1*	**0**	
Number of Participants completed P2	12	14	
Number of Withdrawals	**2** -Personal and family reasons - Hairline fracture while putting on his shoes	**2** -None compliant - Medical recommendation because of development of study unrelated pressure ulcer at the greater trochanter	

*, subjects completed the rest of the measurements per the study protocol. However, based on his knee BMD, we recommended not to complete testing of isometric or isokinetic torques

All participants signed a consent form that was previously approved by the local ethics committee prior to enrollment. Each participant underwent a detailed physical examination, including neurological assessment, and International Standards for Neurological Classification of Spinal Cord Injury (ISNCSCI). Participants with motor complete or incomplete C5-L2 level of injury, the American Spinal Injury Association (ASIA) Impairment Scale (AIS) classification A, B, or C were considered for the trial. Prior to enrollment, both knee extensor muscle groups were electrically stimulated to ensure that all participants did not have lower motor neuron injury or cauda equina injury. Eligibility was determined by visible contraction of the knee extensor and/or movement of leg into extension. A detailed study protocol with the inclusion and exclusion criteria was previously published [13].

## Interventions

### NMES-resistance training (RT) + FES group

Participants randomized into the NMES-RT + FES received an initial 12 weeks of progressive NMES-RT followed by an additional 12 weeks of FES- lower extremity cycling. The training was twice weekly for 24 weeks. Briefly, NMES-RT was applied to the knee extensor muscles via surface electrodes to induce concentric-eccentric actions [3, 12–14]. Two 8 × 10 cm^2^ adhesive carbon electrodes were placed on the skin over the knee extensor muscle group. After placement of the electrodes, NMES parameters were adjusted to a frequency of 30 Hz, biphasic pulses of 450 µs with interpulse interval of 50 µs and amplitude of current sufficient to evoke knee extension. Stimulation of the knee extensor was achieved while subjects remained seated in their wheelchairs through manually increasing the current of NMES unit (Theratouch 4.7; Richmar, Inola, OK) [3, 12–14]. The current was gradually ramped up and then gradually ramped down to ensure moving the leg into knee extension followed by knee flexion. Training was performed twice weekly, separated by at least 48 h. The training session consisted of 4 sets of 10 repetitions that were alternated between the right and left knee extensors and separated by 2 min of rest following each set. The first week was conducted without ankle weights to ensure that the knee extensor muscles would extend the weight of the lower leg against gravity. Once full knee extension was achieved in a sitting position, an increment of 2 lbs. was gradually added per leg on a weekly basis [3, 12–14].

After the initial 12 weeks of NMES-RT, participants underwent P1 measurements in the following week and then the additional 12 weeks of FES- lower extremity cycling was completed, also while seated in their wheelchairs. The wheelchair was firmly docked to the FES-ergometry (RTI-300) via crossed straps, and two wooden bars were inserted underneath the back wheels to prevent any movement during cycling. For FES- lower extremity cycling, rectangular adhesive conductive electrodes were placed on knee extensor, hamstrings, and gluteus maximus muscle groups. Pulse frequency was set at 33.3 Hz, pulse duration at 350 µs and resistance was adjusted every 10 min to maintain a speed of 40–45 revolutions per minute (RPM) [13]. Resistance of the bike was increased in 0.5 Nm increments per 10-min stage over the course of 12 weeks. The progression in resistance was customized based on the subject’s performance riding the FES- lower extremity cycling ergometer over 12 weeks. The fatigue threshold was set at 18 RPM; if RPM fell below 18 RPM, the bike was set to automatically shift from active to passive cycling (cool-down). During the 3-min cool-down period, participants passively cycled with no electrical stimulation. The cool down period was then followed by 5 min of recovery, during which the participant was still connected to the bike but in a complete resting position while having constantly monitored blood pressure and heart rate [13].

### Passive movement training (PMT) + FES group

Passive ROM was applied for 12 weeks prior to FES- lower extremity cycling [13]. A member of the research team supported the leg proximal to the ankle joints and moved it from 90 $^\circ$ knee flexion close to full knee extension. The leg was maintained up for 5 s and returned down for 5 s. The passive movements were repeated in the same fashion described in NMES-RT protocol: 10 reps for the right leg followed by 10 reps for the left leg for total of 4 sets × 10 reps. At the end of initial 12 weeks of PMT, participants underwent P1 measurement and then underwent 12 weeks of FES- lower extremity cycling in the same manner previously described [13].

## Measurements

### Peak isometric & isokinetic torques (PT)

Participants were transferred to the Biodex chair using a ceiling lift and were secured according to manufacturer recommendations using the available straps [3]. The trunk-thigh and thigh-leg angles were set to 85° and 90° respectively. The dynamometer was aligned to the anatomical knee axis, and the lever arm of the knee attachment was seated 2–3 cm superior to the lateral malleolus. Isometric peak torque (i.e., angular velocity of 0 deg.s^−1^) was tested after delivering two different amplitudes (50 mA and 100 mA). The isometric peak torque was tested at different frequencies (10, 30, 50, 80, and 100 Hz) to establish force-frequency curves at either 50 mA or 100 mA at pulse duration of 450µs [3, 20]. Frequencies were randomly delivered, and each frequency was twice and then averaged during data processing. The duration of each electrically evoked contraction lasted for 3–4 s. Furthermore, the difference in peak torque between 100 and 50 mA should provide insight on the effects of training on muscle recruitment [20].

Testing began with the knee in a 90° position. Isometric contractions were maintained for 3–4 s while velocity was set at 0°·s^−1^. The data was sampled at 1000 Hz and processed by a single trained researcher using LabChart (Version 7.3.8; AD Instruments, Colorado Springs, CO). The onset and cessation of isometric torque activity were visually identified by an evaluator blinded to subjects’ group assignment [3]. Isometric PT was calculated by computing the average of a ~ 600 ms window during the plateau quickly following the rise of the torque signal. To account for differences in contraction time, torque-time integral (TTI; Nm.s) was also calculated for the entire contraction [3].

Following isometric peak torque, participants underwent measurements of isokinetic PT (Isok-PT) at different angular velocities (60°, 90°, and 180°·s^−1^; 57.296°·s^−1^ = 1 rad·s^−1^). At least 2 complete concentric knee extensor contractions per limb were elicited via NMES using the same unit. Stimulation parameters were set at 30 Hz, 100 mA and biphasic rectangular pulse of 450µs with electrode placement as described above. Torque-time integral (TTI; Nm/s) was calculated during concentric knee extension at 60°, 90°, and 180°·s^−1^ (respectively Isok-PT-60°, PT-90°, PT-180°) [3].

### Whole thigh and knee extensor cross-sectional area (KE-CSA)

Skeletal muscle CSAs were determined before (baseline), and twice after 12-week interventions (P1 and P2) using a 1.5 Tesla GE magnet (fast spin echo; repetition time, 850–1,000 ms; echo time, 6.7 ms; imaging frequency, 63.8 MHz; echo number, 1; echo train length, 3; flip angle, 90°; field of view, 20 cm; matrix size, 256 × 256) [3, 12–14]. Approximately, 12–15 transaxial images, 0.8 cm thick and 1.6 cm apart, were captured in a supine lying position from the hip to the knee joint (thigh) using a General Electric body array flex coil to measure thigh CSA. Image J software was used to match the images at different time points (BL, P1, P2) using specific anatomical landmarks. Analysis of the whole thigh and knee extensors was performed in a blinded fashion as previously described [3, 12, 13]. Individuals in the lab trained in MRI analysis identified and outlined individual knee extensor muscles for each subject using X-vessel (East Lansing, MI). The software was then used to calculate the whole thigh and knee extensor CSAs (KE CSA) for each slice. The average of across the 12–15 slices was considered as a measure of whole thigh and KE CSA.

Although peak torque and MRI measurements were collected for both left and right legs, we are presenting data only of the left leg only for simplicity purpose. Averaging both legs would likely complicate data processing and interpretation of the results. Additionally, measurements were also not collected on the right leg in two participants because of low bone thresholds as well as history of previous condylar fractures that were unrelated to the study.

### Muscle quality

Muscle quality is a measure of the maximum PT produced during isometric contraction normalized to the amount of muscle CSA: PT (N·m) / CSA (cm^2^). Muscle quality was calculated for the left whole thigh (ST-WT) and KE (ST-KE) CSAs. For simplicity purpose, the isometric PTs elicited at 80 Hz and 100 mA were chosen as representative maximum torques for all participants in both groups [3].

### BMD by dual-energy X-ray absorptiometry (DXA)

Total body and knee scans were measured by DXA using the General Electric Lunar iDXA scanner (GE Lunar Inc, Madison, WI, US) [21–23]. The same technician obtained and analyzed DXA scans at baseline, P1, and P2, to determine regional BMD of the spine, femoral neck, total hip, distal femur, and proximal tibia. After calibration, all participants were placed in a supine lying position on the scanning table with arms internally rotated and palms medially facing. A trapezoid foot positioner held the feet in place and kept the legs from rotating internally, providing minimal overlap between the tibia and fibula. A foam block placed underneath the knees provided knee stability during scanning. Before scanning was initiated, the DXA laser pointer was placed four finger breaths (10 cm) from the distal border of the patella [21–23].

Calculations of BMD were performed for the distal femur and proximal tibia metaphysis. For the proximal tibia metaphysis, a rectangular region of interest was drawn with its height set at 7% of femur length, width set to include only tibial bone, and its proximal edge positioned at the uppermost point of contact between the fibular head and the tibia. For the distal femur metaphysis, a rectangular region of interest was drawn with its height set to match the proximal tibia metaphysis, width to include femoral bone and its lateral end positioned at 13% of femoral length extended up from the lateral condyle [21–24]. In persons with SCI, the coefficient of variabilities of repeated scans for the distal femur and proximal tibia were 5.5% and 3.4%, respectively [25].

### Statistical analyses

All data were tested for normality using the Shapiro–Wilk tests. If normality was not assumed (*P* < 0.05), the examined variable was then log-transformed before conducting any statistical analyses. Outliers were detected using normal Q-Q plots at different time points (BL, P1, P2) for both groups. Outliers were omitted and then the data were then rechecked for normality using the Shapiro–Wilk tests.

Independent *T*-tests were conducted to examine the physical characteristics (age, weight, height, BMI, time since injury) between both groups (NMES-RT + FES and PMT + FES). To account for baseline variabilities, multivariate analysis of covariance (MANCOVA) was conducted to statistically analyze the primary outcome variables (PT, isok-PT, muscle CSA, muscle quality, BMD) of the study. The baseline measurement served as the covariate, both the P1 and P2 measurements served as the dependent variables and the group assignments (NMES-RT + FES vs. PMT + FES) served as a fixed factor.

If the assumptions of MANCOVA were violated, mixed model analysis of variance (MANOVA) was then used to determine whether there was a time effect (baseline, P1 and P2), between group effects or interaction. When appropriate, a Bonferroni post-hoc adjustment for multiple comparisons was performed to control for type II error. Linear regression analyses were used to test the association between the examined variables. Statistical analyses were performed using IBM-SPSS version 29.0 (SPSS, Chicago, IL). Statistical significance was set at alpha level of 0.05 and all values are presented as mean ± SD.

## Results

Thirty-two participants were randomized to NMES-RT + FES (*n* = 16) or PMT + FES (*n* = 16). Physical and SCI characteristics are presented in Table 1. There were no differences in the physical characteristics between groups (*P* > 0.05). Physical characteristics (*n* = 32) were not related to either PT100mA or TTI100mA (*P* > 0.05). Twenty-six participants completed three measurements at BL, P1 and P2. Reasons for withdrawals are listed in Table 1.

Body weight and BMI were related to whole muscle CSA-BL (*n* = 28; weight: r = 0.73, *P* < 0.001 and BMI: *r* = 0.61, *P* < 0.001), knee extensor CSA-BL (*n* = 28; weight: *r* = 0.60, *P* < 0.001 and BMI: *r* = 0.42, *P* = 0.026). Following NMES-RT, average weight lifted on left leg was 11.0 ± 9.0 lbs (0–22 lbs; *n* = 15). At the end of the first 12 weeks, five participants (33%) lifted (0–2 lbs), three participants (20%) lifted (8–14 lbs), and seven participants (47%) lifted (18–22 lbs) in the NMES-RT+FES group.

### Effects of NMES-RT + FES (n = 11–12) vs PMT + FES (n = 13–14) on isometric PT50 mA and PT100mA

In response to an amplitude of 50 mA, neither intervention influenced isometric PT or TT1 at different frequencies (Table 2). A non-significant time effect showed decreases in isometric PT at P1 (13.5–40%) and P2 (16–25%) compared to BL in the NMES-RT + FES group (*n* = 11). A similar non-significant pattern was also detected in the PMT + FES group but only at P2 at 10 Hz (35%) and 30 Hz (10%).

**Table 2:** Isometric peak torque (Nm) and TTI (Nm.s) at 50 mA and 100 mA of participants randomized into either NMES-RT + FES or PMT + FES for 24 weeks

	NMES-RT + FES					PMT + FES				
**Frequencies**	10 Hz	30 Hz	50 Hz	80 Hz	100 Hz	10 Hz	30 Hz	50 Hz	80 Hz	100 Hz
PT50mA-BL (Nm)	2.4 ± 2.1	6.3 ± 6.1	6.6 ± 6.3	8.1 ± 8.4	7.9 ± 6.5	2.5 ± 2.0	6.7 ± 5.5	7.3 ± 6.8	7.8 ± 7.3	7.0 ± 5.6
PT50mA-P1 (Nm)	1.4 ± 1.6	5.4 ± 5.5	5.7 ± 5.4	6.4 ± 6.7	5.6 ± 5.1	2.4 ± 2.6	6.7 ± 7.0	7.6 ± 8.0	9.4 ± 9.7	8.4 ± 7.8
PT50mA-P2 (Nm)	1.8 ± 1.7	5.3 ± 4.9	6.2 ± 5.4	6.8 ± 7.4	6.2 ± 4.7	1.6 ± 1.3	6.0 ± 5.2	7.3 ± 6.1	8.2 ± 5.6	6.7 ± 4.4
PT100mA-BL (Nm)	12.3 ± 8.3	29.1 ± 18.7	30.0 ± 19.0	31.0 ± 18.5	30.4 ± 20.5	15.1 ± 11.4	33.6 ± 23.2	34.4 ± 26.2	37.1 ± 29.3	34.7 ± 24.7
PT100mA-P1 (Nm)	11.3 ± 8.2	37.1 ± 25.0	38.7 ± 25.0	40.8 ± 26.1	38.7 ± 25.0	16.6 ± 13.4	36.1 ± 26.0	37.0 ± 27.0	38.3 ± 26.0	38.2 ± 27.1
PT100mA-P2 (Nm)	12.0 ± 7.7	33.0 ± 18.1	35.2 ± 10.0	36.1 ± 18.8	34.4 ± 18.2	14.4 ± 9.2	35.0 ± 16.0	37.1 ± 18.1	37.6 ± 18.5	36.6 ± 18.1
TTI 50mA-BL (Nm.s)	4.7 ± 4.3	13.8 ± 14.7	16.2 ± 15.1	20 ± 21.3	20.6 ± 20.6	7.5 ± 6.4	16.9 ± 14.9	18.2 ± 17.3	19.5 ± 18.8	17.7 ± 14.1
TTI 50mA-P1 (Nm.s)	3.6 ± 3.8	11.9 ± 12.6	12.3 ± 21.7	15.1 ± 16.8	13.3 ± 13	6 ± 6.7	16.8 ± 18.4	19.4 ± 21.7	22.8 ± 25.3	19.4 ± 19.8
TTI 50mA-P2 (Nm.s)	4.4 ± 3.8	16 ± 17.9	14.8 ± 12.7	15.5 ± 15.5	13.7 ± 10.4	4.0 ± 3.3	15.7 ± 15.5	18.9 ± 18.5	20.5 ± 1	16.6 ± 12.4
TTI 100mA-BL (Nm.s)	26.05 ± 18.31	69.1 ± 47.6	72.3 ± 45.1	72.0 ± 45.2	74.4 ± 51.5	44.0 ± 39.0	93.3 ± 74.1	99.3 ± 83.1	99.9 ± 82.5	93.1 ± 75.5
TTI 100mA-P1 (Nm.s)	23.0 ± 16.1	93.0 ± 64.6	89.7 ± 60.2	96.9 ± 62.3	91.9 ± 61.6	41.0 ± 35.5	90.9 ± 71.1	95.0 ± 77.4	90.8 ± 68.2	95.6 ± 74.7
TTI 100mA-P2 (Nm.s)	27.4 ± 16.7	79.8 ± 47.8	83.5 ± 48.9	88.9 ± 51.4	84.2 ± 47.3	36.0 ± 25.8	91.1 ± 50.8	92.1 ± 56.3	92.1 ± 52.5	88.8 ± 53.2

*PT* isometric peak torque; *BL* baseline; *P1* post-intervention 1; *P2* post-intervention 2; *TTI* torque time integral

In response to an amplitude of 100 mA, a trend (*P* = 0.090) of difference in isometric PT was noted at 80 Hz between NMES-RT + FES (*n* = 12) and PMT + FES (*n* = 13). A similar trend was noted overtime following stimulation with 50 Hz (*P* = 0.08) and 100 Hz (*P* = 0.07) in both groups. However, neither intervention appeared to influence isometric PT or TTI at the remaining frequencies (Table 2).

### Effects of NMES-RT + FES (n = 11–12) vs PMT + FES (n = 13–14) on the difference between isometric PT100mA and PT50 mA

**Fig. 1:**
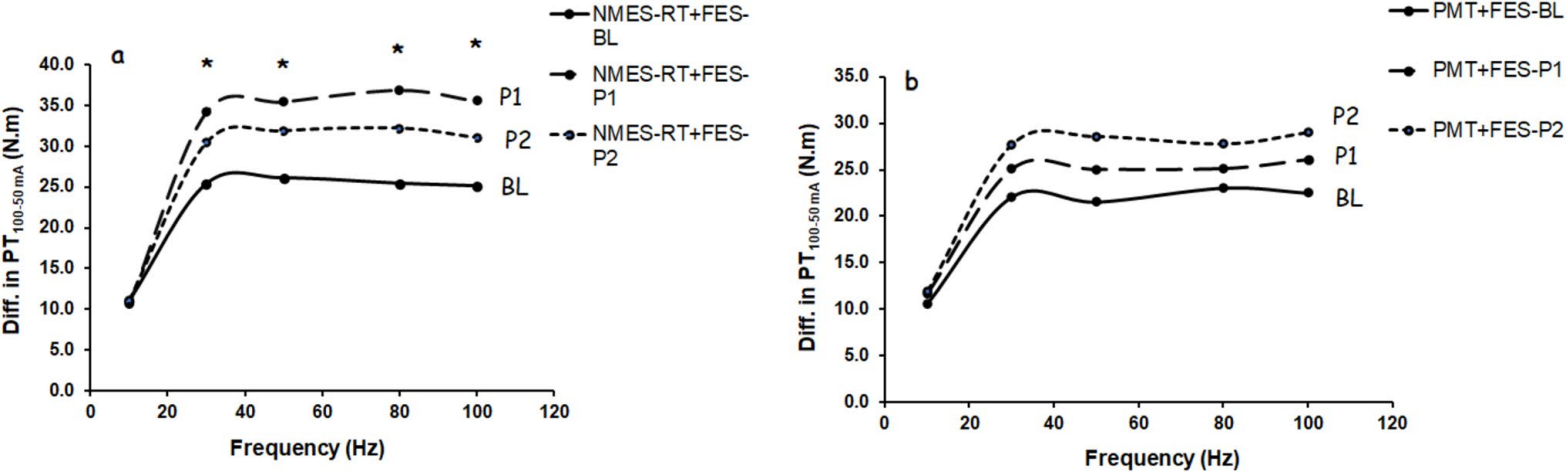
Diff in isometric peak torques (Iso-PT) between 100 and 50 mA as administered at different frequencies at BL, PI and P2 in persons who were randomized into either **a**) NMES-RT + FES or b) PMT + FES. *, time effect at P1 compared to BL in the NMES-RT + FES group at 30 Hz (*P* = 0.035), 50 Hz (*P* = 0.018), 80 Hz (*P* = 0.006) and 100 Hz (*P* = 0.012). Standard deviations were omitted for clarity purposes

Isometric PT50 mA was subtracted from PT100 mA to account for level of muscle recruitment between 50 and 100 mA at different time points (Fig. 1). Normality was assumed after removal of a single outlier data point. MANCOVA revealed that there was a trend [*F* [1, 20] = 4.1; *P* = 0.057; ηp2 = 0.17] toward a difference at a frequency of 80 Hz between NMES-RT + FES and PMT + FES only at P1, but not at P2 (*P* = 0.53). No differences between groups were detected at the remaining frequencies.

Repeated measured ANOVA revealed that there was a time effect in the PT100mA-50mA at 30 Hz [*F* [2, 42] = 5.3; *P* = 009; ηp2 = 0.20], 50 Hz [*F* [2, 42] = 5.3; *P* = 006; ηp2 = 0.22], 80 Hz [*F* [2, 42] = 5.3; *P* = 012; ηp2 = 0.19], and 100 Hz [*F* [2, 42] = 5.3; *P* = 009; ηp2 = 0.20]. Bonferroni pairwise comparisons revealed that time effect occurred in the NMES-RT + FES in P1 compared to BL at 30 Hz (*P* = 0.035), 50 Hz (*P* = 0.018), 80Hz (*P* = 0.006) and 100 Hz (*P* = 0.012), but not in the PMT + FES group (Fig. 1a & b).

### Effects of NMES-RT + FES vs PMT + FES on isokinetic torque

#### Isokinetic TTI 60 deg.s−1

For the isokinetic data, two outliers were detected, and their data were omitted before conducting statistical analyses to assume normal distribution (Table 3). MANCOVA revealed that isokinetic torque was different (*P* = 0.009) between NMES-RT + FES and PMT + FES only at P1, but not at P2 (*P* = 0.58). Repeated measure ANOVA indicated an interaction (*P* = 0.016) between NMES-RT + FES and PMT + FES. A follow-up independent t-test showed a difference (*P* = 0.042) between NMES-RT + FES and PMT + FES at P1.

**Table 3:** Isokinetic TTI (Nm.s) of participants randomized into either NMES-RT + FES or PMT + FES for 24 weeks at angular velocities of 60, 90, and 180 deg/s

	NMES-RT + FES	PMT + FES
Isok-TTI-60 deg/s-BL (Nm.s)	16.0 ± 14.4 (*n* = 14)	20.4 ± 15.7 (*n* = 15)
Isok-TTI-60 deg/s-P1 (Nm.s)	28.0 ± 18.0 #, x (*n* = 12)	17.0 ± 11.0 (*n* = 14)
Isok-TTI-60 deg/s-P2 (Nm.s)	29.0 ± 12.0 (*n* = 10)	23.0 ± 12.0 (*n* = 12)
Isok-TTI-90 deg/s-BL (Nm.s)	13.0 ± 14.6 (*n* = 14)	19.3 ± 18.0 (*n* = 15)
Isok-TTI-90 deg/s-P1 (Nm.s)	22.5 ± 17.0 x! (*n* = 12)	12.5 ± 8.8 (*n* = 14)
Isok-TTI-90 deg/s-P2 (Nm.s)	22.0 ± 8.6 (*n* = 10)	16.2 ± 10.7 (*n* = 12)
Isok-TTI-180 deg/s-BL (Nm.s)	9.3 ± 15.0 (*n* = 14)	14.2 ± 14.5 (*n* = 15)
Isok-TTI-180 deg/s-P1 (Nm.s)	16.0 ± 12.4#, x! (*n* = 12)	8.5 ± 6.4 (*n* = 14)
Isok-TTI-180 deg/s-P2 (Nm.s)	23.4 ± 6.3 (*n* = 10)	10.1 ± 7.8 (*n* = 12)

Isok: isokinetic; TTI: Torque-time integral (Nm.s); BL: baseline; P1: post-intervention 1; P2: post-intervention 2

^#^, between group effect; x: interaction between groups; x!: a trend towards interaction

#### Isokinetic TTI 90 deg.s^−1^

Repeated measure ANOVA indicated a trend towards interaction (*P* = 0.06) between NMES-RT + FES and PMT + FES without between (*P* = 0.3) group differences.

#### Isokinetic TTI 180 deg.s^−1^

MANCOVA revealed that isokinetic torque was different (*P* = 0.003) between NMES-RT + FES and PMT + FES only at P1, but not at P2 (*P* = 0.29). Repeated measure ANOVA indicated a trend towards interaction (*P* = 0.09) between NMES-RT + FES and PMT + FES.

### Effects of NMES-RT + FES vs PMT + FES on muscle CSA

MANCOVA indicated that left whole thigh P1 [*F* [2, 20] = 9.1; *P* = 0.007] and left KE [*F* [2, 20] = 15.5; *P* = 0.001] muscle CSAs were different, but not at P2 (whole thigh: *P* = 0.63 and KE:* P* = 0.81). Diff in whole thigh revealed a within time effect [*F* [1, 20] = 0.36; *P* = 0.001; ηp2 = 0.42]. Pairwise comparisons indicated a between group difference at P1 of whole thigh P1-BL of 10.1 cm^2^; *P* < 0.001 without changes at P2 (Table 4).

**Table 4:** Left whole thigh muscle and knee extensor CSAs as well as muscle quality of participants randomized into either NMES-RT + FES (*n* = 12) or PMT + FES (*n* = 13) for 24 weeks

		NMES-RT + FES (*n* = 12)	PMT + FES (*n* = 13)
Whole Muscle CSA (cm^2^)	Whole Muscle CSA BL	92.6 ± 25.5	86.2 ± 22.4
	Whole Muscle CSA P1	103.7 ± 26.6^#^	88.3 ± 24.8
	Whole Muscle CSA P2	107.5 ± 27.9	98.9 ± 25.2
Knee extensor CSA (cm^2^)	Knee Extensor CSA BL	39.6 ± 10.4	38.4 ± 13.6
	Knee Extensor CSA P1	47.8 ± 12.0^#^	39.57 ± 14.8
	Knee Extensor CSA P2	49.0 ± 13.3	47.2 ± 15.4
Muscle quality-whole muscle (Nm/cm^2^)	ST Whole Muscle BL	0.33 ± 0.17	0.32 ± 0.19
	ST Whole Muscle P1	0.39 ± 0.23	0.33 ± 0.18
	ST Whole Muscle P2	0.33 ± 0.20	0.41 ± 0.29
Muscle quality-knee extensor (Nm/cm^2^)	ST KE-BL	0.79 ± 0.42	0.70 ± 0.39
	ST KE P1	0.84 ± 0.47	0.70 ± 0.41
	ST KE P2	0.72 ± 0.39	0.69 ± 0.33

^#^, between group differences at P1 for whole thigh muscle CSA (*P* = 0.007) and for knee extensor CSA (*P* < 0.001)

Repeated measure ANOVA indicated a within time effect [*F* [1, 21] = 42.1; *P* = 0.001; ηp2 = 0.67] and interaction [*F* [1, 21] = 22.6; *P* = 0.001; ηp2 = 0.52] the difference of muscle CSA of KE. Pairwise comparisons indicated that 7.14 cm^2^ was gained in KE muscle CSA (*P* = 0.001) as result of NMES-RT + FES, but not in the other group (Table 4).

### Ankle weights

**Fig. 2:**
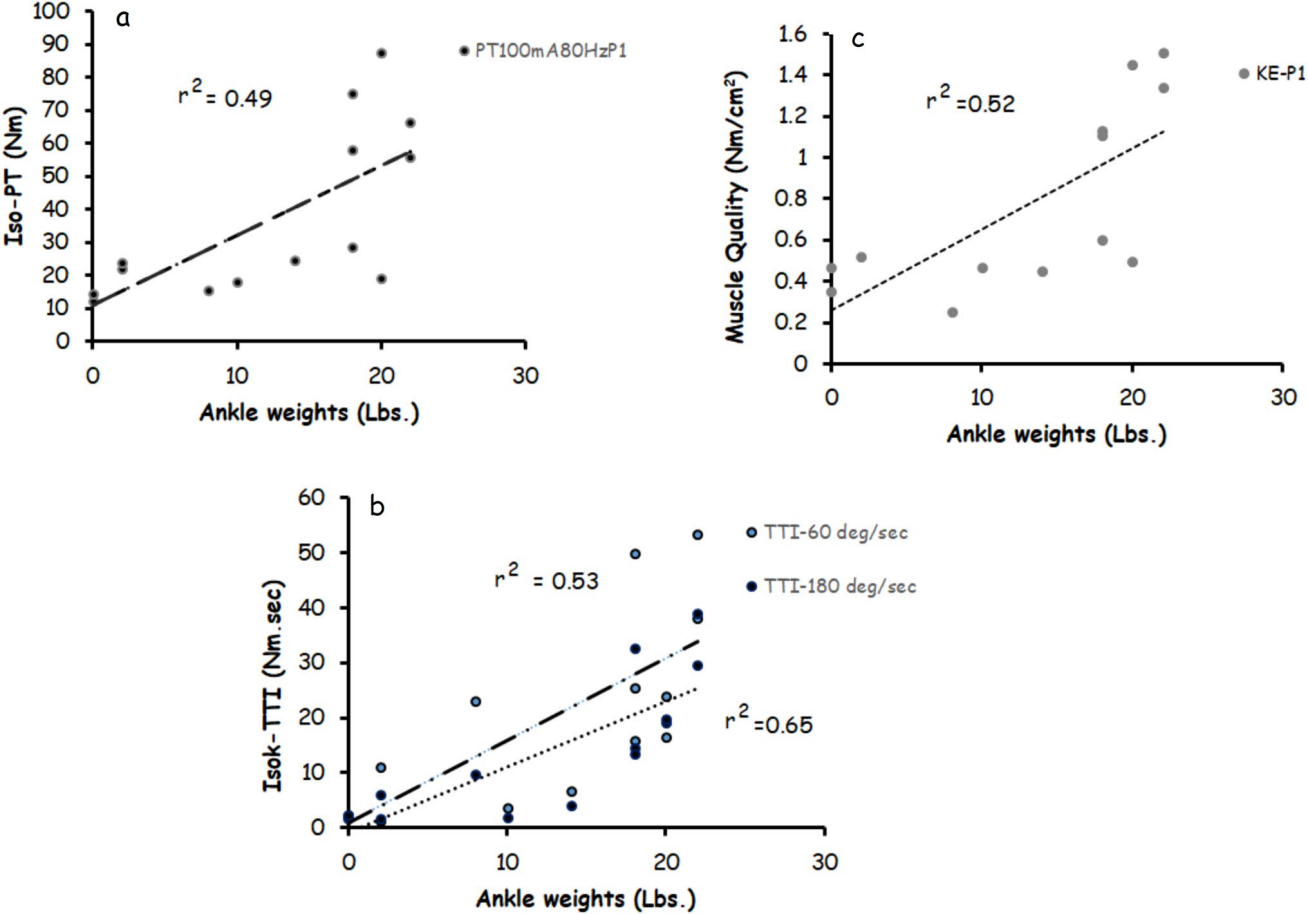
Relationships between ankle weights and **a**) isometric peak torque (Iso-PT) applied at an amplitude of 100mA and frequency of 80 Hz during post-intervention 1; **b**) isokinetic- torque time integral (isoK-TTI) captured at angular velocities of 60 deg/s or 180 deg/s; and **c**) muscle quality as determined by isometric PT at 80 Hz and 100 mA relative to the KE muscle CSA

The amount of ankle weight showed a positive trend with whole thigh (*r* = 0.48; *P* = 0.08; *n* = 14) and KE (*r* = 0.51, *P* = 0.06; *n* = 14) muscle CSAs (Fig. 2). The relationships between ankle weights and whole thigh (*r* = 0.65; *P* = 0.016) and KE (*r* = 0.61; *P* = 0.026) muscle CSAs became significant after controlling for body weight.

Figure 2 presents the relationships between ankle weights and isometric PT-100mA at different frequencies [30 Hz: *r* = 0.68, *P* = 0.007, 50 Hz: *r* = 0.71, *P* = 0.005, 80Hz: *r* = 0.70, *P* = 0.005 and 100 Hz: *r* = 0.65, *P* = 0.01; Fig. 2a), isokinetic TTI at different angular velocities [60 deg/s: *r* = 0.77, *P* = 0.001, 90 deg/s: *r* = 0.73, *P* = 0.003, and 180 deg/s: *r* = 0.81, *P* = 0.001; Fig. 2b], ST [whole thigh: *r* = 0.72, *P* = 0.005 and KE: *r* = 0.72, *P* = 0.006; Fig. 2c) and muscle recruitment as determined by the difference in PT between 100 and 50 mA [30 Hz: *r* = 0.68, *P* = 0.007, 50 Hz: *r* = 0.70, *P* = 0.005, 80Hz: *r* = 0.69, *P* = 0.006 and 100 Hz: *r* = 0.64, *P* = 0.01; Fig. 2d).

### Effects of NMES-RT + FES vs PMT + FES on muscle quality and BMD

The were no effects of either interventions on muscle quality at P1 or P2 (Table 4). There was a within group time effect on distal femur BMD [*F* [2] = 4.9; *P* = 0.01; ηp2 = 0.17]. Pairwise comparisons indicated a decrease in BMD following PMT + FES at P2 [mean diff: −0.064 g/cm^2^; 7%; *P* = 0.03]. No other changes in BMD were noted following either intervention (Table 5).

**Table 5:** BMD (g/cm^2^) as measured by DXA of the SCI participants who randomized into either NMES-RT + FES or PMT + FES for 24 weeks

	NMES-RT + FES	PMT + FES
Dist. Femur BMD BL	0.82 ± 0.21 (*n* = 12)	0.86 ± 0.22 (*n* = 14)
Dist. Femur BMD P1	0.79 ± 0.22 (*n* = 12)	0.81 ± 0.20 (*n* = 14)
Dist. Femur BMD P2	0.78 ± 0.20 (*n* = 12)	0.80 ± 0.18 (*n* = 14)*
Prox. Tibia BMD BL	0.97 ± 0.29 (*n* = 12)	0.97 ± 0.25 (*n* = 14)
Prox. Tibia BMD P1	0.94 ± 0.30 (*n* = 12)	0.97 ± 0.24 (*n* = 14)
Prox. Tibia BMD P2	0.95 ± 0.27 (*n* = 12)	0.96 ± 0.23 (*n* = 14)
Hip BMD BL	0.89 ± 0.49 (*n* = 12)	0.79 ± 0.16 (*n* = 14)
Hip BMD P1	0.91 ± 0.57 (*n* = 12)	0.87 ± 0.32 (*n* = 14)
Hip BMD P2	0.87 ± 0.38 (*n* = 12)	0.79 ± 0.15 (*n* = 14)
Pelvis BMD BL	0.96 ± 0.29 (*n* = 15)	1.00 ± 0.42 (*n* = 16)
Pelvis BMD P1	0.89 ± 0.19 (*n* = 15)	0.91 ± 0.16 (*n* = 16)
Pelvis BMD P2	0.86 ± 0.17 (*n* = 15)	0.93 ± 0.14 (*n* = 16)
Spine BMD BL	1.32 ± 0.18 (*n* = 15)	1.32 ± 0.13 (*n* = 16)
Spine BMD P1	1.31 ± 0.22 (*n* = 15)	1.39 ± 0.33 (*n* = 16)
Spine BMD P2	1.30 ± 0.21 (*n* = 15)	1.40 ± 0.35 (*n* = 16)
Total BMD BL	1.19 ± 0.11 (*n* = 15)	1.23 ± 0.13 (*n* = 16)
Total BMD P1	1.17 ± 0.13 (*n* = 15)	1.22 ± 0.15 (*n* = 16)
Total BMD P2	1.16 ± 0.12 (*n* = 15)	1.22 ± 0.14 (*n* = 16)

BMD: bone mineral density; Dist.: distal; prox.: proximal; BL: baseline; P1: post-intervention 1; P2; post-intervention 2

*, pairwise comparison indicated time effect compared to BL (*P* = 0.03)

Regression models at P1 indicated that the whole thigh CSA-P1, Iso-PT at 80 Hz-P1 and ST- whole thigh -P1 (*n* = 20) accounted for 45% in the variance of the proximal tibia BMD (*r*^2^ = 0.45;* P* = 0.020). The model revealed that the whole thigh CSA was trending towards being a significant predictor of proximal tibia BMD (*β* = −1.2; *P* = 0.08). Additionally, whole thigh CSA explained 54% of the variance in proximal tibia (*n* = 15; *P* = 0.046) in a model that encompassed ankle weights (*β* = −0.2; *P* = 0.5) and whole thigh CSA (*β* = 0.83; *P* = 0.02).

## Discussion

The major goal of the current trial was to examine the combined effects of two different paradigms of electrical stimulation exercises (NMES-RT and FES- lower extremity cycling) on muscle quality and subsequently bone health in persons with chronic SCI. Isometric and isokinetic torques as well as muscle CSA were captured to measure muscle quality. The major findings indicated that NMES-RT + FES increased components of muscle quality as determined by greater isometric PT at 80 Hz, greater isokinetic torques at 60 deg/s and 180 deg/s as well as evoking muscle hypertrophy after 12 weeks of NMES-RT at P1 compared to PMT. However, muscle quality as determined by the ratio of maximum force to muscle CSA was not changed. The increases in isometric peak torque, isokinetic torque, and muscle hypertrophy were primarily attributed to increasing the magnitude of lifted ankle weights. Despite the lack of changes in muscle quality, NMES-RT + FES maintained BMD at the distal femur compared to PMT + FES.

### Implications of the trial to the existing knowledge

Persons with SCI experience detrimental effects on their musculoskeletal system including muscle atrophy, diminished torque production, premature fatigue, and development of neurogenic osteoporosis [6–9]. There is a dynamic interplay between muscle quality and bone health that has been previously explored in the general population [18, 19]. The current study was undertaken to test the hypothesis that improvement in muscle quality or its components may positively enhance bone adaptations. We have designed the trial to administer the minimum frequency per week (twice weekly) that is unlikely to induce changes in BMD as measured by DXA. Previously, a similar frequency was adopted for 9 months and did not induce any differences in BMD [26]. The duration of the entire trial was only limited to 6 months because it is typically associated with a small decrease in the rate of BMD loss [7].

Mechanical loading is considered the most appropriate intervention for increasing BMD similar to jogging, jumping activities, etc. However, this is not an option for persons with SCI. Offering RT is well recognized for increasing lean mass and muscle hypertrophy [12, 13]. This may provide the necessary loading while performing FES- lower extremity cycling in persons with SCI. This has been previously observed in post-menopausal women and following long-duration spaceflight [28, 29]. In postmenopausal women, the combined RT with aerobic training was regarded as a better approach than RT only [28].

In persons with SCI, mechanically loading the muscle could be administered through either open-kinematic chain exercise (NMES-RT) or closed-kinematic chain program (FES- lower extremity cycling) [3, 12, 13, 17]. NMES-RT was regarded as the most effective rehabilitation tool to robustly evoke muscle hypertrophy in persons with SCI [30]. The open-kinematic chain exercise increases bone strain [14]; whereas the close-kinematic chain enhances compressive forces on the paralyzed lower extremities; especially distal femur and proximal tibia [15–17]. Unlike previous studies, we adopted both opened and closed kinematic chain exercises via applications of NMES-RT and FES- lower extremity cycling, respectively. Despite the short intervention duration, NMES-RT + FES maintained BMD at the distal femur compared to the PMT + FES group. Previously, a period of 9–12 months was recommended to study bone changes in response to electrical stimulation exercise after SCI [31, 32]. However, progress in software capabilities offered utility to study regional BMD adaptations in persons with SCI [22, 23].

Previous studies used various forms of closed-kinematic chain exercise such as passive standing [33], treadmill training [34], FES-lower extremity cycling [27, 35] and compressive loading of the paralyzed soleus muscles to attenuate bone loss after SCI [36]. Today, the addition of an open-kinematic chain exercise not only enhances muscle quality but may also increase the mechanosensitivity of the muscles and the abundance of different signaling pathways [37, 38], which may upregulate positive bone adaptations. This muscle-bone interplay is likely to be enhanced by the production of myokines [39, 40]. Unlike mechanical loading exerted by the muscles on the bone, myokines are likely to exert a direct effect on bone metabolism especially after SCI [40]. Several myokines have been identified in the field similar to myostatin and irisin that have been shown to facilitate signaling pathways necessary to enhance bone strength and mass [40]. Therefore, conditioning the muscles prior to mechanically loading the paralyzed bones may further enhance bone formation after SCI.

The combined exercise paradigm may also serve as the basis of future home-based clinical trials for a longer duration in conjunction with other pharmacological interventions such as vitamin D supplementation or testosterone treatment. We have recently noted that 97% of persons with SCI do not meet the recommended daily guidelines of dietary vitamin D intake [22]. Addition of vitamin D to the proposed paradigm may boost the effect of exercise on trabecular microarchitectures at the distal femur and proximal tibia. Another important determinant of bone mass is the circulating level of testosterone [23], which appears to be influenced by fat mass [23]. Therefore, the current paradigm of exercise is likely to increase lean mass and reduce fat mass [12, 13]; which may augment the actions of circulating testosterone on bone mass after SCI [23].

### Muscle quality and BMD after SCI

In the current trial, we hypothesized that enhancing muscle quality via training would influence BMD in persons with SCI. Most of the clinical trials suggested a duration greater than 6 months and up to 5 sessions per week to induce positive bone changes after SCI [31, 32, 35]. This is not clinically feasible and finding strategies to induce faster bone adaptations is highly recommended for this population. We, therefore, proposed that conditioning the muscle to enhance muscle quality or its components may result in faster bone adaptations despite limited frequency and duration of the intervention. The findings suggested that enhancing the components of muscle quality via NMES-RT may attenuate BMD loss in the distal femur following 12 weeks of FES- lower extremity cycling. This was not the case in the PMT + FES group.

It is possible to assume that ankle weights used during NMES-RT were associated with increasing components of muscle quality (Fig. 2), which allowed FES- lower extremity cycling to mechanically stress the bones in the second phase of the trial. Sixty-seven percent of our sample was capable of lifting ankle weights equal to or greater than 8 lbs. over the course of 12 weeks. Previously, participants lifted an average of 20 lbs when NMES-RT was combined with testosterone treatment over the course of 16 weeks [3]. A previous trial indicated that FES- lower extremity cycling with lower cycling cadence generated 75% greater average torque than normal cycling cadence [35]. The lower cycling cadence resulted in improvement in bone health as characterized by decreasing biomarkers of bone turnover and enhancing trabecular microarchitectures [35]. Therefore, evoking muscle hypertrophy during NMES-RT resulted in increasing the evoked torque generated during FES- lower extremity cycling and attenuated bone loss compared to PMT + FES.

The study offers insights regarding the importance of carefully screening the participants before enrollment in the NMES-RT program. In the current trial, 33% participants who failed to lift more than 2 lbs. ranged from C6-T12. This means that the level of injury cannot provide guidance on the appropriate candidate for the NMES-RT program. We previously developed a screening tool by using the number of repetitions as well as the amplitude of the current to examine muscle and BMD early in the training program [41]. Participants who failed to achieve less than 70% of 40 repetitions (4 sets × 10 reps per leg) as well as using current amplitude greater than 100 mA had lower muscle mass and knee BMD than their counterparts with SCI [3]. Furthermore, higher-responders with SCI to NMES-RT have greater body weight and fat mass as well as greater upregulation of signaling pathways responsible for evoking muscle hypertrophy compared to low-responders with SCI [42]. Therefore, considering these factors may help to properly assign candidates into different groups when studying muscle quality and BMD in persons with SCI.

### Limitations

The major limitation was the study was not primarily powered to test the current hypothesis. The study was initially powered to determine the effects of both paradigms of training on cardio-metabolic risk factors [13]. Furthermore, our results are limited to persons with SCI who are using wheelchairs as a primary mode of mobility. We did not include persons with incomplete SCI who are actively mobile or using other weightbearing devices for ambulation. Compared to our earlier reports [3, 12, 14], we included persons with incomplete AIS C. Difficulties in reaching statistical significance in several of the examined variables is attributed to the wide heterogeneous nature of the studied population as result of injury classification, time since injury and other physical characteristics such as differences in gender and ethnicity. This may have resulted in masking effects on the outcome variables of the study. We have limited the results to the left side for simplicity purpose in data analysis. Previously, presenting both sides separately did not yield additional information. Additionally, the ankle weight lifted was similar between both the left (11 lbs) and the right (11 lbs) legs following NMES-RT. We have also introduced different frequencies (10–100 Hz) in a random order for each participant and maintained similar order for the same participant at BL, PI and P2 [20]. The order of the frequencies (i.e.30 Hz before 100 Hz or 100 Hz before 30 Hz) may have generated differences in isometric PT and challenged the outcomes of training [43–45]; however, this hypothesis has yet to be tested after SCI. Future trials may also need to consider examining only 100 mA at two frequencies (30 and 80 Hz) to reduce potential neuromuscular fatigue that may result from repetitive stimulation. The use of DXA to evaluate regional changes following 3-month interval need to be considered in lieu of the existing guidelines that recommends bone evaluation every 12 months to detect meaningful changes [46].

## Conclusions

The major finding indicated that enhancement in components of muscle quality may potentially attenuate bone loss in persons with SCI. Compared to PMT + FES, NMES-RT + FES resulted in muscle hypertrophy and maintained BMD at the distal femur over 24 weeks of training. Muscle hypertrophy was accompanied with increases in isokinetic torques of the trained knee extensors as well as the level of muscle recruitment without impacting muscle quality. The magnitude of ankle weights was likely to be the key predictor of muscle CSA, torque production, and muscle quality in persons with SCI. Future directions should consider a prospective home-based longitudinal randomized clinical trial with a longer duration via secured telehealth system to test the current hypothesis with a larger sample size. The current findings have future implications as researchers and clinicians are aiming to improve muscle quality and bone health to facilitate standing and walking after SCI.

## Data Availability

Data will be available upon contacting the corresponding author and after obtaining necessary approvals from our research office.
